# Design and Micro-Nano Fabrication of a GaAs-Based On-Chip Miniaturized Bandpass Filter with Intertwined Inductors and Circinate Capacitor Using Integrated Passive Device Technology

**DOI:** 10.3390/nano12030347

**Published:** 2022-01-21

**Authors:** Jian Chen, Bao-Hua Zhu, Shan Yang, Wei Yue, Dong-Min Lee, Eun-Seong Kim, Nam-Young Kim

**Affiliations:** 1 Radio Frequency Integrated Circuit Center, Kwangwoon University, Wolgye-Dong, Nowon-Ku, Seoul 139-701, Korea; cnjacob@kw.ac.kr (J.C.); zhuwangwhy@kw.ac.kr (B.-H.Z.); shanpymy@kw.ac.kr (S.Y.); yuewei@kw.ac.kr (W.Y.); thxks@kw.ac.kr (D.-M.L.); 2 NDAC Centre, Kwangwoon University, 20 Kwangwoon-ro, Wolgye-Dong, Nowon-Ku, Seoul 139-701, Korea

**Keywords:** gallium arsenide, air-bridge structure, bandpass filter, capacitor, inductor, integrated passive device, micro-nano fabrication

## Abstract

In this study, we propose a miniaturized bandpass filter (BPF) developed by combining an approximate circular (36-gon) winding inductor, a circinate capacitor, and five air-bridge structures fabricated on a gallium arsenide (GaAs) substrate using an integrated passive device (IPD) technology. We introduced air-bridge structures into the outer metal wire to improve the capacitance per unit volume while utilizing a miniaturized chip with dimensions 1538 μm × 800 μm (0.029 λ_0_ × 0.015 λ_0_) for the BPF. The pattern was designed and optimized by simulating different dimensional parameters, and the group delay and current density are presented. The equivalent circuit was modeled to analysis various parasitic effect. Additionally, we described the GaAs-based micro-nano scale fabrication process to elucidate the proposed IPD technology and the physical structure of the BPF. Measurements were conducted with a center frequency of 1.53 GHz (insertion loss of 0.53 dB) and a 3-dB fractional bandwidth (FBW) of 70.59%. The transmission zero was located at 4.16 GHz with restraint of 35.86 dB. Owing to the benefits from its miniaturized chip size and high performance, the proposed GaAs-based IPD BPF was verified as an excellent device for various S-band applications, such as satellite communication, keyless vehicle locks, wireless headphones, and radar.

## 1. Introduction

Passive devices such as filters, balancers, mixers, and power dividers have been widely studied in the past few years owing to the importance of radio frequency (RF) and microwaves in wireless communication systems. Low-pass, high-pass, band-pass, and band-stop filters, are the most common filter types in the field of microwave filters. Among them, band-pass filters (BPFs) have been extensively studied and widely used in the RF front-end of receivers and transmitters considering they constitute RF/microwave integrated circuits and systems [1,2]. While early RF research extensively studied microstrip filters considering they were low cost, easy to process, lightweight, and possessed multi-component integration capabilities, they exhibited insufficient miniaturization and higher losses. Therefore, design and manufacturing technologies that require higher accuracy, smaller size, lower loss, lower cost, and mass production have been explored by researchers [3].

In the past few decades, several manufacturing technologies, such as monolithic microwave integrated circuits (MMICs), microelectromechanical systems (MEMSs), low-temperature co-fired ceramics (LTCCs), and high-temperature superconductors (HTSs), have been widely studied to meet the growing market demand [4]. The LTCC technology embeds several passive components (such as resistors, capacitors, and inductors) in ceramics through punching, grouting, and printing, which reduces the size of the entire module. For example, see Ref. [5], where the insertion loss is 2.4 dB and the physical size is 6.9 × 39.9 mm^2^. Additionally, it allows ceramics to be sintered with highly conductive materials (silver, copper, and gold) that exhibit low resistance and low conductor loss at high frequencies. However, modules that require heat dissipation must have a heat sink, which complicates processing. Moreover, LTCC technology has higher requirements for the size and characteristics of the processed plates given that ceramics shrink after firing [6,7]. HTSs have developed rapidly in recent years considering their low surface resistance, which is comparable to ordinary conductive metals. Additionally, passive components based on HTSs have almost no signal loss during transmission, which makes them attractive. Furthermore, their low-loss characteristics help fabricate relatively compact and complex designs of microwave passive components. For example, see Ref. [8], where the insertion loss is 0.2 dB and the physical size is 20.8 × 16.15 mm^2^ [9]. However, their low temperature requirements increase the cost and complexity, thereby limiting their development and popularity. MEMSs can largely integrate several passive components on a substrate, resulting in a microstructure of 20 μm to 1 mm, which reduces the size and weight of the device. Furthermore, MEMSs are widely used considering they can be mass produced while reducing manufacturing costs. For example, see Ref. [10], where the insertion loss is 5.2 dB and the physical size is 5.2 × 12.3 mm^2^. However, the stability of their dynamic range and capability of power handling needs to be improved [11]. MMICs have gained wide attention due to advantages such as higher integration, smaller size, lighter weight, higher reliability of wire bonding, and lower cost of a single IC during mass production. Additionally, introducing III-V compounds such as gallium nitride (GaN), gallium arsenide (GaAs), and indium antimonide (InSb) can enhance the functionality and reliability of MMICs. For example, see Ref. [12], where the insertion loss is 0.95 dB and the physical size is 0.0369 × 0.0428 λ_0_^2^. However, they exhibit low power capability and are incapable of making design changes during manufacturing, which restricts the development of MMICs to a certain extent [13,14].

Integrated passive device (IPD) techniques have rapidly developed, benefiting from the advantages of integration ability, compact size, and small parasitic effects compared to standard discrete systems [15,16]. Additionally, IPDs can be packaged with active integrated circuits or other IPDs in electronic system components, or stacked in the third dimension (3D), which further improves their integration. The quality factor (Q-factor) is given in this study because it is the most important indicator to evaluate the quality of BPF suffering from ohmic loss, eddy current, and electromagnetic (EM) interference when spiral inductors appear in the design [17]. Based on our previous research, the IPD technology can provide a better Q-factor to a certain extent [18,19,20]. Additionally, this study explores an IPD-based BPF using a GaAs substrate, which is a very important semiconductor material in the medical, communications, and military fields. Electrons move 5-10 times faster in GaAs than in silicon. Furthermore, it has a higher breakdown voltage compared to silicon and glass. Therefore, a semiconductor made of GaAs has the characteristics of high energy band, high cut-off frequency and high power [21,22]. The wide application was profit from its low field mobility, low parasitic tendency, and good isolation between devices [23,24,25,26,27]. The proposed IPD-based BPF comprises an approximately circular (36-gon) spiral intertwined inductor with five air-bridges on the outside and a non-crossing circinate capacitor at the center. Section 2 describes the design, optimization and micro–nano fabrication of the BPF, in which Section 2.1 introduces the design, optimization, equivalent circuit modeling and analysis, and Section 2.2 presents the micro–nano fabrication process to clarify the realization of the complex physical structure of the device in detail. Section 3 demonstrates the measured result of the proposed BPF using a vector network analyzer (VNA), and satisfactory agreement of the measured and simulated results is achieved. Lastly, we demonstrate the advantages of this research by comparing it with the published BPFs.

## 2. Design and Micro-Nano Fabrication

### 2.1. Design, Optimization and Analysis

**Figure 1:**
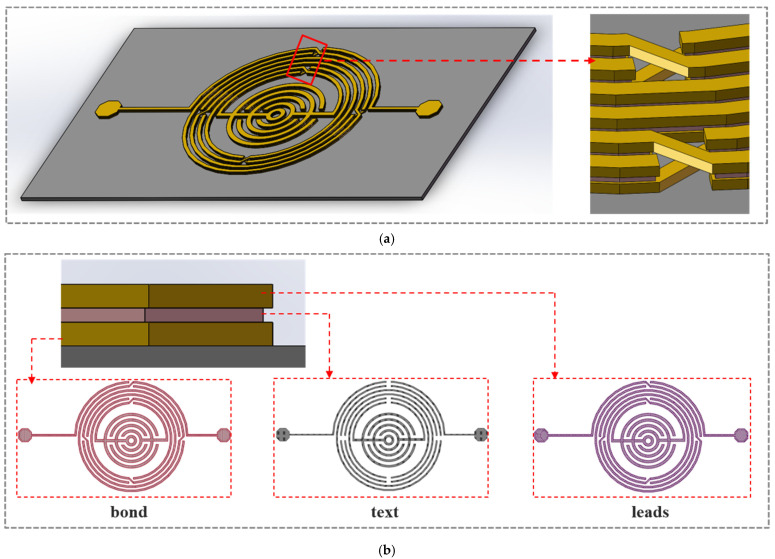
Pattern design of the proposed BPF: (**a**) stereo view of the BPF on a GaAs substrate and enlarged view of the air-bridges; (**b**) side view and three metal layers (leads, text, and bond).

The design, simulation, optimization, and verification of the proposed GaAs-based BPF were performed using the Agilent Advanced Design System 2016 (ADS, Keysight Technologies Inc., Santa Rosa, CA, USA). Figure 1a shows a stereo view of the proposed BPF, and an enlarged view illustrates the air-bridge structures. A circinate capacitor is located at the center of an approximate circular (36-gon) spiral inductor. The side view in Figure 1b shows three laminated conductor layers comprising 90% Cu and 10% Au, named bond, text, and leads in ADS (bottom to top).

#### 2.1.1. Filter Analysis and Optimization Based on Various Parameters

**Figure 2:**
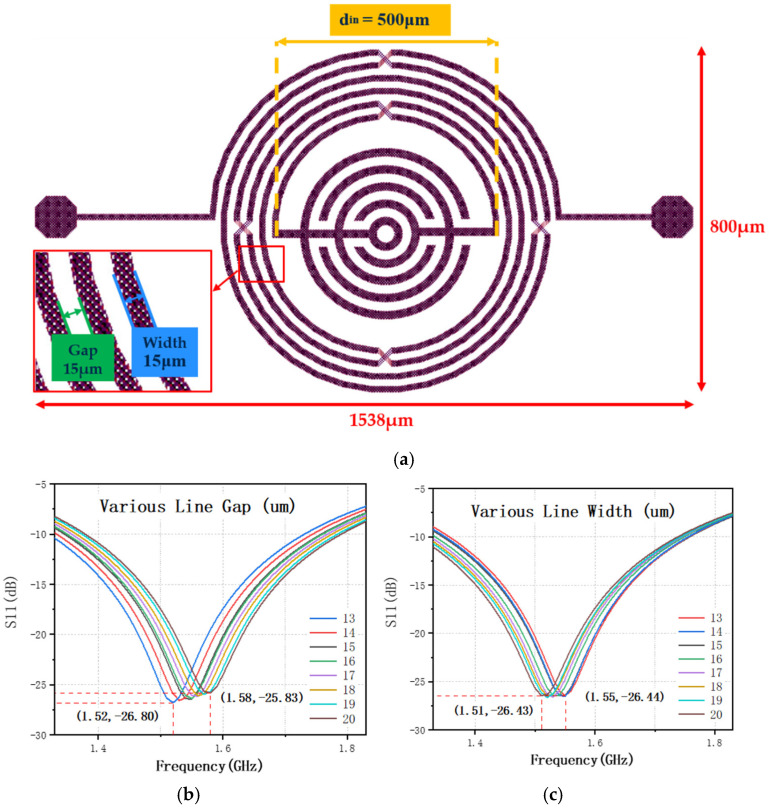
Simulation results on the influence of the two parameters on the S-parameter and center frequency: (**a**) top view of the proposed IPD BPF layout with the markers of metal line width and gap; (**b**) simulation results with different line widths; (**c**) simulation results with different line gaps.

To explore the influence of different dimensional parameters on the resonance characteristics of the proposed BPF, we varied the metal line width (15 μm) and metal line gap (15 μm) for simulation and analysis, as shown in Figure 2a. Table 1 and Table 2 summarize the adjustments to line gap and line width, respectively, and a total of 16 detailed dimension information were utilized for the analysis. The line width of the text layer in the middle is always 4 μm narrower than that of the bond and leads layers.

**Figure 3:**
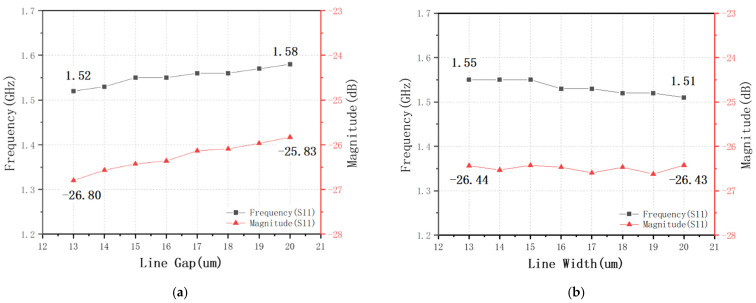
Shifting liner analysis of the center frequency and magnitude with different layout pa-rameters: (**a**) line gap and (**b**) line width.

As shown in Figure 2, the changes in physical parameters yielded different results. Figure 2b,c shows the influence of different metal line gaps and metal line widths on the resonant frequency and magnitude, respectively. To further explore the linear changes exhibited by the resonance, we conducted a linear analysis based on the experimental data, as shown in Figure 3.

Figure 2 and Figure 3 shows that the center frequency and magnitude increase as the gap increases, whereas the center frequency decreases as the width increases while the magnitude is barely moved. Combined with theoretical analysis, it can be seen that this result is reasonable. The center frequency increases with the increase of the line gap, because the coupling effect between the metal lines weakens and the capacitance becomes smaller; the center frequency decreases with the increase of the line width (gap remains unchanged), because the length of the metal line increases increased inductance [28,29].

**Figure 4:**
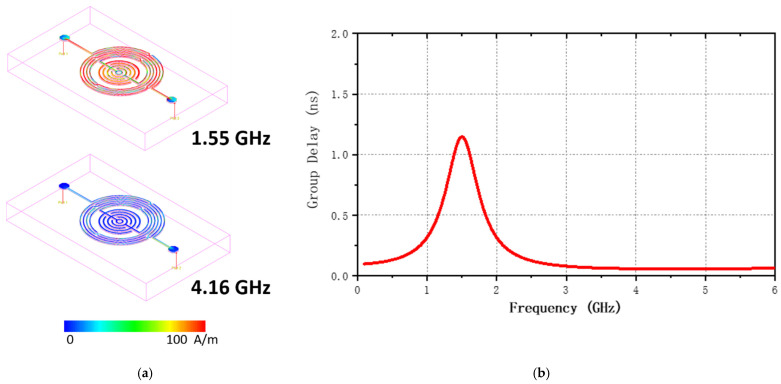
Results of optimization: (**a**) current densities of the passband and stopband; and (**b**) group delay.

We decided to maintain the design of 15 μm gap and 15 μm width (center frequency of 1.55 GHz, amplitude of −26.43 dB) after the above simulation and analysis, and the current density and group delay simulation are performed to verify the optimized performance of this design. As shown in Figure 4a, the current density at the frequency of the resonance point, that is, the passband, was significantly higher than that at the stopband. Additionally, the group delay is used to judge the distortion of the signal when it passes through the filtering system, that is, the smaller the group delay, the better the ability of the signal to maintain its shape. In this study, we used ADS to simulate the group delay directly. The group delay of the entire design is always lower than 1.25 ns, the time delay of the signal passing through the amplitude envelope of each sine component of the device under test is small, indicating that the BPF has a good ability to maintain the signal shape, as shown in Figure 4b [30,31].

#### 2.1.2. Equipment Circuit Analysis

**Figure 5:**
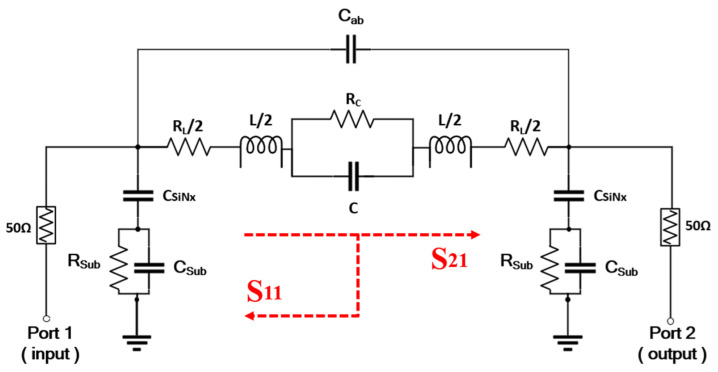
Equipment circuit model of the proposed BPF.

Owing to the fact that achieving an ideal circuit without loss is impossible, we propose an equivalent circuit diagram of the π-type LC BPF while ignoring some smaller feed-on capacitance and loss impedance values, as shown in Figure 5. The lumped-element model mainly comprises capacitances, inductances, and substrate-associated parasitic capacitances.

The loss resistance in the inductor (L) and capacitor (C) including the resistance caused by proximity effect, are denoted by R_L_ and R_C_, respectively [4]. R_Sub_ and C_Sub_ represent the resistance and capacitance associated with the substrate whereas C_SiNx_ represents the capacitance of the SiN_x_ passivation layers. Considering the skin effect, the current density is largest near the surface of the conductor; hence, the above parameters can be expressed [32,33] as:

(1) RL=ρlwδ1−etδ,

(2) $$C_{\mathrm{Sub}}=\frac{1}{2}{\mathrm{lwC}}_{0},$$

(3) $$R_{\mathrm{Sub}}=\frac{2}{{\mathrm{lwG}}_{0}},$$

(4) $$C_{{\mathrm{SiN}}_{x}}=\frac{1}{2}\mathrm{lw}\frac{\varepsilon_{{\mathrm{SiN}}_{x}}}{d_{{\mathrm{SiN}}_{x}}},$$

where ρ is the electrical resistivity, *δ* is the skin depth of the metal trip, and w, t, and l are the, width, thickness and length of the metal strip, respectively. C_0_ and G_0_ are the capacitance and conductivity per unit area of the GaAs substrate, respectively. d_SiNx_ and ε_SiNx_ are the thickness and dielectric constant of the SiN_x_ passivation layer, respectively. However, the L and C are still dominate the resonant frequency comparing with the various parasitic effects analyzed above. The value of the inductance formed by the outer intertwined metal wire is given [34] as:

(5) $$L=\frac{{\mu n}^{2}d}{2}\left[\ln\left(\frac{2.46}{\eta }\right)+0.2\eta^{2}\right],$$

(6) $$\eta =\frac{d_{\mathrm{out}}-d_{\mathrm{in}}}{d_{\mathrm{out}}+d_{\mathrm{in}}},$$

(7) $$d=\frac{\left(d_{\mathrm{out}}+d_{\mathrm{in}}\right)}{2},$$

where μ is the magnetic permeability, η is the number of turns of the inductor, d is the average diameter of the inner and outer rings of the inductor, and the value of η and d are approximately 0.25 and 665, respectively. 

The parasitic effect of the bridge is related to the overlap of three metal layers, so the thickness of the air-bridge and the dielectric constant of free space are important parameters, which are represented by t_ab_ and ε_0_ (overlap area) respectively. Since the length of the air-bridge is much smaller than that of the entire differential inductor, the resistance and inductance of air-bridge can be ignored and only the main capacitance effect is considered, which can be expressed [35] as:

(8) $$C_{\mathrm{ab}}=\frac{\varepsilon_{0}\left(\mathrm{overlap}\;\mathrm{area}\right)}{t_{\mathrm{ab}}}.$$

By combining Figure 2a and Figure 5, it can be seen that the air-bridges are connected in series. This means that the introduction of series air-bridge reduces the parasitic capacitive effect and increases the inductance and Q-factor of differential inductor. The capacitance can be obtained using the linear function between the radius of the innermost circle and the capacitance of the concentric pattern. Given that the capacitance value is only affected by the material properties of the fixed-width ring and the distance between two adjacent rings [36,37], a capacitor model is established in ADS to simulate and optimize the capacitance effect between the metal layer and the ground. Meanwhile the resistor R_C_ is introduced in optimize process to construct the embedded center circinate capacitor model while considering the ohmic loss. To simplify the calculation of the above parameters caused by the complex structure of the central capacitor, simulated Y-parameters are introduced to calculate the capacitance and resistance, as depicted in Equations (9) and (10) [38].

(9) $$C\left(\mathrm{pF}\right)=\frac{1\times 10^{12}\times \mathrm{imag}\left(Y_{11}\right)}{2\pi f},$$

(10) $$R_{C}\left(\Omega \right)=\mathrm{real}\left[\frac{1}{Y\left(1,1\right)}\right].$$

**Figure 6:**
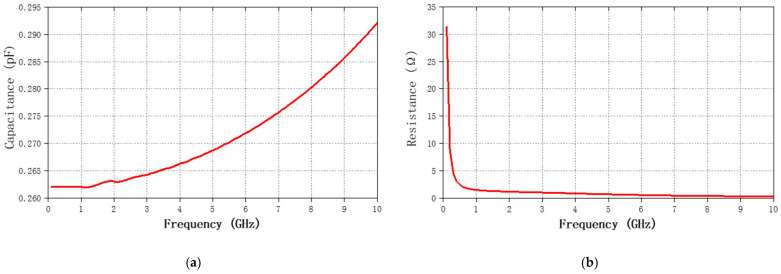
Simulation results of the capacitance and resistance for optimization: (**a**) Simulated capacitance value; (**b**) Simulated resistance value.

Figure 6 shows the optimized frequency-dependent simulation results of the capacitance and resistance of the center capacitor. As shown in Figure 6a, the capacitance value is relatively stable with some insignificant differences in the working frequency band of the proposed BPF. In addition, we simulated the Q-factors of L and C, which were 38.12 and 317.24, respectively. By combining the embedded capacitor and external inductor, the center frequency f_0_ of the π-type LC model-based design can be expressed [39] as:

(11) $$f_{0}=\frac{1}{2\pi \sqrt{\mathrm{LC}}}.$$

### 2.2. Micro-Nano Fabrication

**Figure 7:**
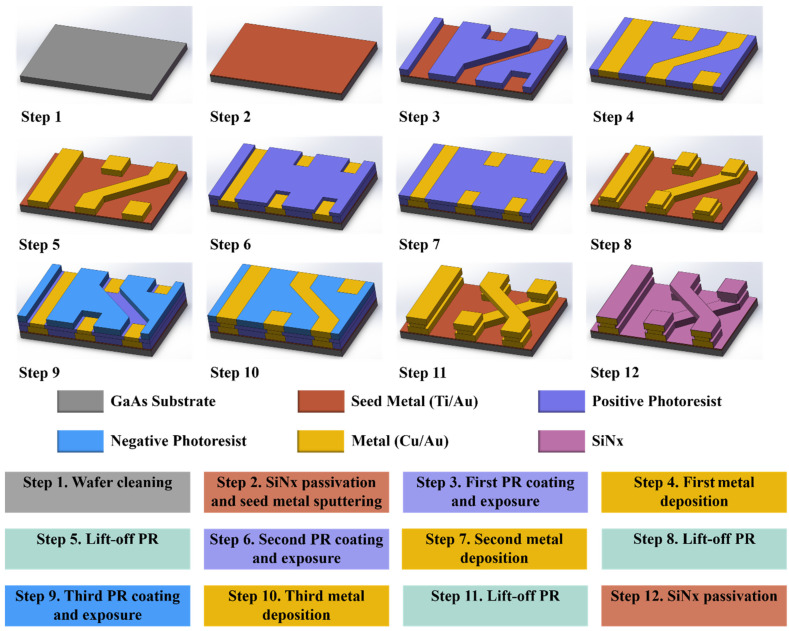
Fabrication of the proposed GaAs-based integrated passive device (IPD).

Figure 7 shows a diagram of the 12-step micro-nano fabrication process of the IPD technology for proposed BPF. The device was manufactured and cut on a 6-inch GaAs substrate. The air-bridge structure, which is the most complicated part of this device, was considered as a representative in Figure 7 to intuitively explain the entire microfabrication process. First, an acetone bath, isopropanol (IPA), and deionized (DI) water were used to treat and eliminate ionic contaminants, organic impurities, and natural chemical oxides on the surface of the GaAs wafer, as shown in Step 1. Next, plasma-enhanced chemical vapor deposition (PECVD) was exploited to deposit a 200 nm thickness SiNx passivation layer (relative permittivity: 7.5, loss tangent: 0.002) in a chamber environment of the mixture of SiH_4_ and NH_3_ (ratio of 1:19), with temperature of 250 °C, pressure of 1200 mTorr, gas flow of 2000 sccm, and an RF power of 100 W, to obtain a flat wafer surface. Subsequently, a seed metal layer with 20 nm Ti and 80 nm Au was formed through the sputtering process to enhance the adhesion between the passivation layer and first metal layer, as shown in Step 2. Subsequently, the positive photoresist was coated onto the preliminarily processed wafer using a spin-coater, and the layout of the first metal layer was defined by the exposure and development process, as shown in Step 3. The first metal layer (bond layer) with 4.5 and 0.5 μm thick Cu and Au, respectively, was electroplated on the wafer surface in an environment of 5.0 × 10^−6^ mTorr pressure, 0.5 Å/s minimum deposition rate, and 10 kV electron energy (Step 4). Then, the photoresist was peeled off in a lift-off machine with an environment of acetone/IPA/DI water mixture for 90 s (Step 5). Subsequently, the positive photoresist was spin-coated on the wafer again, and the second exposure and development process were applied to define the layout of the middle metal layer, as shown in Step 6. The second metal layer (test layer) with 1.6 and 0.2 μm thick Cu and Au, respectively, was electroplated on the wafer surface (Step 7) using the same deposition process as the bottom metal layer. Likewise, the remaining photoresist was eliminated using the lift-off machine to obtain the results shown in Step 8. Subsequently, a 6.8 μm thick positive photoresist was spin-coated on the wafer surface until it was flushed with the second metal layer. Then, a 5 μm thick negative photoresist was spin-coated on the positive photoresist and was allowed to expose and develop to define the layout of the top metal layer, as shown in Step 9. It should be noted that because the line width of the top metal layer is wider than that of the middle layer metal, two types of photoresists must be used simultaneously to perfectly expose the shape of the top metal layer. Lastly, the third metal layer (lead layer) with 4.5 and 0.5 μm thick Cu and Au, respectively, was electroplated on the wafer surface (Step 10) using the same deposition process as the previous metal layer. Similarly, the remaining photoresist was peeled off using the lift-off machine for 90 s, a sufficient time to ensure the complete stripping of the PR without residue, to obtain the results shown in Step 11. Subsequently, a 300 nm SiNx passivation layer was deposited on the entire surface (Step 12) to protect the device from moisture and oxidation. Ultimately, the fabricated resonator was mounted on the PCB via polishing, cutting, and wire bonding processes to measure the RF performance of the manufactured devices. Table 3 summarizes the details of the technologies and metals used in the manufacturing process.

## 3. Results and Discussion

**Figure 8:**
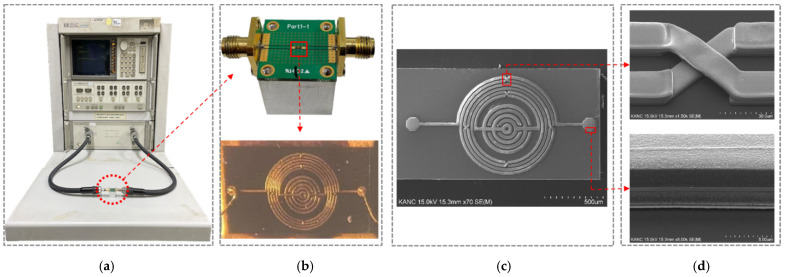
(**a**) Measurement setup (VNA); (**b**) BPF fixed on an aluminum cube and the top view of the fabricated product; (**c**) top view of the SEM image; (**d**) enlarged view of air-bridge structure and cross-section of the three metal layers.

We designed and simulated an IPD resonator using ADS, and compared the test data with the simulation data. Furthermore, we measured and recorded the transmission and reflection parameters of the product using an Agilent 8510C vector network analyzer (VNA), as shown in Figure 8a. The aluminum cube (2^3^ cm^3^) acts as a GND to reduce noise, and the PCB was mounted on it. The two ports of the PCB are connected to the VNA using subminiature version A (SMA) connectors. The chip was wire-bonded on the PCB, as shown in Figure 8b. As shown in Figure 8c, the size of the product is 1538 μm × 800 μm, which is marked in a scanning electron microscope (SEM) image. As seen in Figure 8d, the enlarged view of air-bridge structures and a cross-section clearly shows its three-layer structure.

**Figure 9:**
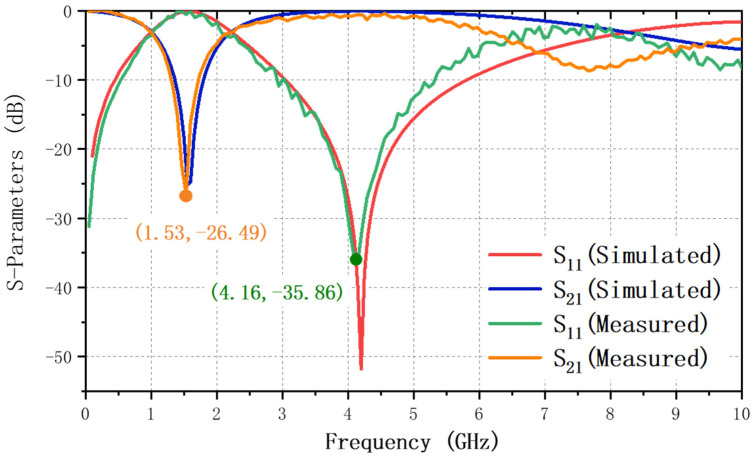
Simulation and measurement results of the S11 and S21 parameters.

Figure 9 compares the simulation and measurement parameters of the IPD, showing a good consistency. In the simulated result, the center frequency is located at 1.55 GHz with the insertion loss and return loss being 25.17 dB and 0.08 dB, respectively. The center frequency was measured at 1.53 GHz with a 3-dB passband of 0.99–2.07 GHz and a fractional bandwidth of 70.59%. Only 0.02 GHz frequency shift and 1.32 dB return loss variation, respectively, are acceptable manufacturing errors. The transmission zero with a frequency and magnitude of 4.16 GHz and −35.86 dB, respectively, is located on the right side of the passband. The insertion loss is 0.53 dB, return loss is 26.49 dB, and the Q-factor is 49.29. Table 4 compares the proposed IPD BPF with four researched BPFs to demonstrate that the proposed device exhibits relatively small chip size, wide fractional bandwidth, and good insertion and return losses.

Table 5 compares the present study with other works using various manufacturing technologies, thereby demonstrating the merits of a wide passband and smaller dimensions of the proposed GaAs-based IPD BPF.

## 4. Conclusions

In this study, we proposed a micro–nano scale BPF comprising an approximate circular (36-gon) winding inductor and a circinate capacitor using the GaAs-based IPD technology. The equivalent circuit model was established by considering various capacitive and inductive parasitic effects. The three-layer BPF was fabricated in 12 steps using thin-film and photolithography processes. The fabricated BPF had a miniaturized overall size of 1538 μm × 800 μm (0.029 λ_0_ × 0.015 λ_0_). The insertion loss is as low as 0.53 dB, and the 3-dB FBW is as wide as 70.59%, which shows that the measured results share a relatively good consistency with the theoretical prediction and simulation. Furthermore, the proposed BPF can be employed in modern communication systems owing to its high performance and miniaturized size. Additionally, it can also be used as a biosensor due to its quick RF response time and non-contact detection. However, considering that this study did not investigate the lifecycle and compatibility of the device in practical applications, it will be explored and ameliorated in our future research. Furthermore, its selectivity is limited considering it is a low-order device, which will also be improved and studied in our next research to promote the development of the IPD technology in practical applications.

## Figures and Tables

**Table 1:** Dimensional information of the proposed IPD BPF line gap adjustment.

	Bond Layer (Line Width 15)	Test Layer (Line Width 11)	Leads Layer (Line Width 15)
**Line Gap**	13	17	13
	14	18	14
	15	19	15
	16	20	16
	17	21	17
	18	22	18
	19	23	19
	20	24	20

All data in the table are in micrometers.

**Table 2:** Dimensional information of the proposed IPD BPF line width adjustment.

	Bond Layer (Line Gap 15)	Test Layer (Line Gap 19)	Leads Layer (Line Gap 15)
**Line Width**	13	9	13
	14	10	14
	15	11	15
	16	12	16
	17	13	17
	18	14	18
	19	15	19
	20	16	20

All data in the table are in micrometers.

**Table 3:** Manufacturing techniques used in the IPDs process.

Fabrication Objective	Technique	Material
Passivation layer	PECVD	SiNx
Photo resistor	Spin-coating	Negative/positive PR
PR removal	Lift-off	Acetone
Seed metal	Sputtering	Ti/Au
Metal layer	Electroplating	Cu/Au
Via	ICP etching	SF_6_/O_6_

**Table 4:** Performance comparison of the proposed BPF and published BPFs.

Ref.	Fabrication Process	Circuit Area *	Passband (GHz)	3-dB Fractional Bandwidth (%)	Insertion Loss (dB)	Return Loss (dB)
[40]	Glass-IPD	<1.00 mm^2^ (0.018 λ_0_ × 0.009 λ_0_)	2.6	49.62	0.6	30
[41]	Si-IPD	0.72 mm^2^ (0.024 λ_0_ × 0.024 λ_0_)	2.4	33.33	2.3	10
[42]	Si-IPD	3.9 mm^2^ (0.039 λ_0_ × 0.037 λ_0_)	1.7	≈17.72	2.54	12
[43]	Glass-IPD	1.69 mm^2^ (0.019 λ_0_ × 0.019 λ_0_)	2.1	≈8.6	3.2	22
This work	GaAs-IPD	1.23 mm^2^ (0.029 λ_0_ × 0.015 λ_0_)	1.53	70.59	0.53	26.49

* λ_0_ is the guided wavelength of the operation frequency.

**Table 5:** Comparisons between this study and other works using various manufacturing technologies.

Ref.	Manufacturing Technology	Fractional Bandwidth (%)	Insertion Loss (dB)	Return Loss (dB)	Passband (GHz)	Circuit Area
[44]	Microstrip	13.3	1.1	>20	0.975	0.094 λ_0_ × 0.08 λ_0_
[8]	HTS	66.7	0.2	19	1.5	20.8 × 16.15 mm^2^ 0.318 λ_0_ × 0.247 λ_0_
[45]	HTCC	5.5	1.8	>15	2.25	0.182 λ_0_ × 0.156 λ_0_
[5]	LTCC	12.5	2.4	15	2.4	6.9 × 39.9 mm^2^
This work	GaAs IPD	70.59	0.53	26.49	1.53	1.538 × 0.8 mm^2^ (0.029 λ_0_ × 0.015 λ_0_)

## References

[1] Liang J.G., Wang C., Kim N.Y. (2017). Dual-band ultra-wideband bandpass filter with eight-resonant modes and quad-transmission zeros employing synchronous-quasi-resonance. Radioengineering.

[2] Liang J.G., Wang C., Kim N.Y. (2018). Implementation of ultra-wideband bandpass filter with modularized design based on synchronous-quasi-resonance and double-curved-route. IET Microw. Antennas Propag..

[3] Li Y., Wang C., Kim N.Y. (2015). Design of very compact bandpass filters based on differential transformers. IEEE Microw. Wireless Compon. Lett..

[4] Chen J., Wang Z.J., Zhu B.H., Kim E.S., Kim N.Y. (2020). Fabrication of QFN-packaged miniaturized GaAs-based bandpass filter with intertwined inductors and dendritic capacitor. Materials.

[5] Zhang X.Y., Dai X., Kao H.L., Wei B.H., Cai Z.Y., Xue Q. (2014). Compact LTCC Bandpass Filter With Wide Stopband Using Discriminating Coupling. IEEE Trans. Microw. Theory Technol..

[6] Wang K.X., Liu X.F., Li Y.C., Lin L.Z., Zhao X.L. (2017). LTCC filtering rat-race coupler based on eight-line spatially-symmetrical coupled structure. IEEE Access.

[7] Dubey M., Suri N., Khanna P.K. (2013). Optimization of shrinkage and surface-roughness of LTCC tape. Int. J. Res. Eng. Technol..

[8] Tsukamoto O., Ogawa J. (2007). Perspectives of elementary technologies for AC power applications of high temperature superconductors. J. Mater. Process. Technol..

[9] Liu H., Rao L., Xu Y. (2016). Design of high-temperature superconducting wideband bandpass filter with narrow-band notch resonators for radio telescope application. IEEE Trans. Appl. Supercond..

[10] Nishino T., Kitsukawa Y., Hangai M., Lee S., Soda S., Miyazaki M., Naitoh I., Konishi Y. Tunable MEMS hybrid coupler and L-band tunable filter. Proceedings of the 2009 IEEE MTT-S International Microwave Symposium Digest.

[11] Lei D., Wang T., Cao D., Fei J. (2016). Adaptive dynamic surface control of mems gyroscope sensor using fuzzy compensator. IEEE Access.

[12] Shen G., Che W., Feng W., Shi Y., Shen Y. (2020). Low insertion-loss MMIC bandpass filter using lumped-distributed parameters for 5G millimeter-wave application. IEEE Trans. Compon. Packag. Manuf. Technol..

[13] McCulloch M.A., Melhuish S.J., Piccirillo L. (2014). Enhancing the noise performance of monolithic microwave integrated circuit-based low noise amplifiers through the use of a discrete preamplifying transistor. J. Astron. Telescopes Instrum. Syst..

[14] Robertson I.D., Lucyszyn S. (2001). RFIC and MMIC Design and Technology.

[15] Zuo C., He C., Cheng W. Hybrid filter design for 5G using IPD and acoustic technologies. Proceedings of the 2019 IEEE International Ultrasonics Symposium (IUS).

[16] Liu L., Kuo S.M., Abrokwah J. (2007). Compact harmonic filter design and fabrication using IPD technology. IEEE Trans. Compon. Packag. Technol..

[17] Talwalkar N.A., Yue C.P., Wong S.S. (2005). Analysis and synthesis of on-chip spiral inductors. IEEE Trans. Electron. Devices.

[18] Kim E.S., Kim N.Y. (2018). Micro-fabricated resonator based on inscribing a meandered-line coupling capacitor in an air-bridged circular spiral inductor. Micromachines.

[19] Wang C., Kim N.Y. (2012). Analytical optimization of high-performance and high-yield spiral inductor in integrated passive device technology. Microelectron. J..

[20] Li Y., Wang C., Kim N.Y. (2015). A high performance compact Wilkinson power divider using GaAs-based optimized integrated passive device fabrication process for LTE application. Solid-State Electron..

[21] Weitzel C.E. (1995). Comparison of SiC, GaAs, and Si RF MESFET power densities. IEEE Electron Device Lett..

[22] Afsar M.N., Button K.J. (1983). Precise Millimeter-Wave Measurements of Complex Refractive Index, Complex Dielectric Permittivity and Loss Tangent of GaAs, Si, SiO_2_, A1_2_O_3_, BeO, Macor, and Glass. IEEE Trans. Microw. Theory Tech..

[23] Liu Z., Wang Q., Song C., Cheng Y. (2017). Similarity-based difference analysis approach for remaining useful life prediction of GaAs-based semiconductor lasers. IEEE Access.

[24] Nitesh R.S., Rajendran J., Ramiah H., Manaf A.A. (2018). A 700 MHz to 2.5 GHz cascode GaAs power amplifier for multi-band pico-cell achieving 20 dB Gain 40 dBm to 45 dBm OIP3 and 66% Peak PAE. IEEE Access.

[25] Niu H., Gao S., Yue W., Li Y., Zhou W., Liu H. (2020). Highly morphology-controllable and highly sensitive capacitive tactile sensor based on epidermis-dermis-inspired interlocked asymmetric-nanocone arrays for detection of tiny pressure. Small.

[26] Kim N.Y., Adhikari K.K., Dhakal R., Chuluunbaatar Z., Wang C., Kim E.S. (2015). Rapid sensitive and reusable detection of glucose by a robust radiofrequency integrated passive device biosensor chip. Sci. Rep..

[27] Dhakal R., Wang C., Kim E.S., Kim N.Y. (2015). Complex permittivity characterization of serum with an air-bridge enhanced capacitor for quantifiable detection of glucose. Appl. Phys. Lett..

[28] Maeda M. (1972). An analysis of gap in microstrip transmission lines. IEEE Trans. Microw. Theory Tech..

[29] Djordjevic A.R., Sarkar T.K. (1994). Closed-form formulas for frequency-dependent resistance and inductance per unit length of microstrip and strip transmission lines. IEEE Trans. Microw. Theory Tech..

[30] Quan C.H., Wang Z.J., Lee J.C., Kim E.S., Kim N.Y. (2019). A Highly Selective and Compact Bandpass Filter with a Circular Spiral Inductor and an Embedded Capacitor Structure Using an Integrated Passive Device Technology on a GaAs Substrate. Electronics.

[31] Dam H.H., Cantoni A., Nordholm S., Teo K.L. (2008). Variable digital filter with group delay flatness specification or phase constraints. IEEE Trans. Circuits Syst. II Express Briefs.

[32] Aryan N.P. (2014). Design and Modeling of Inductors, Capacitors and Coplanar Waveguide at Tens of GHz Frequencies.

[33] Haobijam G., Palathinkal R.P. (2013). Design and Analysis of Spiral Inductors.

[34] Mohan S.S., del Mar Hershenson M., Boyd S.P., Lee T.H. (1999). Simple Accurate Expressions for Planar Spiral Inductances. IEEE J. Solid-State Circuits.

[35] Li Y., Yao Z., Fu X.Q., Li Z.M., Shan F.K., Wang C. (2017). The development of differential inductors using double air-bridge structure based on integrated passive device technology. Solid-State Electron..

[36] Chen T., Bowler N. (2013). Design of Interdigital Spiral and Concentric Capacitive Sensors for Materials Evaluation. AIP Conf. Proc..

[37] Chen T., Bowler N. (2010). Analysis of a Concentric Coplanar Capacitive Sensor for Nondestructive Evaluation of Multi-Layered Dielectric Structures. IEEE Trans. Dielectr. Electr. Insul..

[38] Matthaei G.L., Chinn G.C., Plott C.H., Dagli N. (1992). A simplified means for computation for interconnect distributed capacitances and inductances. IEEE Trans. Comput.-Aided Des. Integr. Circuits Syst..

[39] Hong J.S. (2011). Microstrip Filters for RF/Microwave Applications.

[40] Sitaraman S., Sukumaran V., Pulugurtha M.R., Wu Z., Suzuki Y., Kim Y., Sundaram V., Kim J., Tummala R.R. (2017). Miniaturized bandpass filters as ultrathin 3-D IPDs and embedded thinfilms in 3-D glass modules. IEEE Trans. Compon. Packag. Manuf. Technol..

[41] Pan J., Wang H., Tian G., Cao L., Yu D. (2016). Design of a compact silicon-based integrated passive band-pass filter with two tunable finite transmission zeros. Microelectron. J..

[42] Wong K.W., Mansour R.R., Weale G. (2017). Reconfigurable bandstop and bandpass filters with wideband balun using IPD technology for frequency agile applications. IEEE Trans. Compon. Packag. Manuf. Technol..

[43] Wu S.M., Hsu R.F., Yu P.H. (2013). Signal integrity and electromagnetic broadband packaging model extraction of full differential bandpass filter on IPD with BGA packaging. Prog. Electromagn. Res..

[44] Lu D., Barker N.S., Tang X.H. (2017). Miniaturized two-pole lumped BPF with four controllable TZs using multiple coupling paths. IEEE Microw. Wirel. Compon. Lett..

[45] Zhu Y.Y., Yang Y.J., Chen J.X. (2018). High-performance bandpass filter using HTCC stepped-impedance resonators. IET Microw. Antennas Propag..

